# Review: structure and modifications of arabinogalactan proteins (AGPs)

**DOI:** 10.1186/s12870-023-04066-5

**Published:** 2023-01-20

**Authors:** Agata Leszczuk, Panagiotis Kalaitzis, Joanna Kulik, Artur Zdunek

**Affiliations:** 1 Institute of Agrophysics, Polish Academy of Sciences, Doświadczalna 4, 20-290 Lublin, Poland; 2 Department of Horticultural Genetics and Biotechnology, Mediterranean Agronomic Institute of Chania, Chania, P.O. Box 85, 73100 Chania, Greece

**Keywords:** Arabinogalactan proteins, Cell wall, Development, Growth, Plant tissue, Plasma membrane, Proteoglycans, Ripening

## Abstract

The aim of this report is to provide general information on the molecular structure and synthesis of arabinogalactan proteins (AGPs) in association to their physiological significance. Assessment of genetic modifications of the activity of enzymes involved in the AGP biosynthesis is an efficient tool to study AGP functions. Thus, P4H (prolyl 4 hydroxylase) mutants, GLCAT (β-glucuronosyltransferase) mutants, and GH43 (glycoside hydrolase family 43) mutants have been described. We focused on the overview of AGPs modifications observed at the molecular, cellular, and organ levels. Inhibition of the hydroxylation process results in an increase in the intensity of cell divisions and thus, has an impact on root system length and leaf area. In turn, overexpression of *P4H* genes stimulates the density of root hairs. A mutation in *GLCAT* genes responsible for the transfer of glucuronic acid to the AGP molecule revealed that the reduction of GlcA in AGP disrupts the substantial assembly of the primary cell wall. Furthermore, silencing of genes encoding GH43, which has the ability to hydrolyze the AGP glycan by removing incorrectly synthesized β-1,3-galactans, induces changes in the abundance of other cell wall constituents, which finally leads to root growth defects. This information provides insight into AGPs as a crucial players in the structural interactions present in the plant extracellular matrix.

## Background - cell wall proteins

The plant cell wall is a dynamic structure associated with key processes in the plant life cycle. It provides mechanical strength to cells, participates in their enlargement and differentiation, protects them against abiotic and biotic stress, constitutes a barrier preventing the entry of pathogens, and is responsible for the morphogenesis and architecture of the whole plant [1, 2]. All these roles are related to the cell wall complex structure. The primary cell wall consists mainly of three classes of polysaccharides, i.e. cellulose, hemicelluloses, pectins, and a lower amount of proteins, so-called cell wall proteins (CWPs) [2, 3]. The CWPs are structurally heterogeneous. Moreover, their biological activity and conformation are altered by post-translational modifications (PTMs) during transport through the secretory pathway [4–7].

CWPs are represented by hydroxyproline (Hyp)-rich *O*-glycoproteins (HRGPs superfamily), which include arabinogalactan proteins (AGPs). In addition to AGPs, which are the most highly glycosylated proteins, the HRGP family also comprises slightly arabinosylated proline-rich proteins (PRPs) and highly arabinosylated extensins (EXTs) [4]. Each subfamily belonging to HRPG has common features, e.g. repetitive consensus sequences that define the way of their glycosylation according to the Hyp-*O*-glycosylation code and distinguishing features such as arabinogalactosylated glycomodule variables, the presence of hydroxyproline-based *O*-glycosylation sites, the presence of functional domains, and the ability to be anchored to the cell membrane with the GPI anchor [8, 9]. Despite the available knowledge about the widespread occurrence and functions of HRGPs, still little is known about how particular members of the family work and achieve their functions [10]. There is evidence that HRGPs mechanically enhance the cell wall as scaffolds for the deposition of other molecules and are involved in sealing off walls [4].

## Definition of arabinogalactan proteins (AGPs)

AGPs are highly glycosylated glycoproteins characterized by high heterogeneity due to their complicated structure, which is composed of different protein backbones, and the different content of carbohydrate moieties [11–15]. Initially, AGPs were defined as a group of molecules meeting basic criteria: the ability to bind to the β-Yariv reagent [16, 17] and the presence of a Hyp-rich core-protein backbone connected with carbohydrate chains [18]. For many years, the specific reaction between AGPs and Yariv reagent was an essential criterion in the recognition of AGPs, and the ability of AGP binding by the reagent facilitates the isolation of AGPs from plant tissue. The β-Yariv reagent is a synthetic phenylglycoside [1,3,5-tri(p-glycosyloxyphenylazo)-2,4,6-trihydroxybenzene], but the structure of AGP to which the β-Yariv reagent binds has not yet been determined. It is known that the carbohydrate portion of AGP is recognized, and the interaction mainly requires β-1,3-galactan chains (longer than five residues) and a protein moiety. In addition, the binding capacity of the reagent is influenced by the substitution range of β-(1,6)-galactan [13]. Interestingly, β-Yariv is able to self-aggregate up to about 305 units in an aqueous solution, and the size of the aggregates affects the interaction with AGP. Thus, the β-Yariv reagent can perturb the action of AGP *in planta.* This attribute is widely used in AGP functional research for investigations of physiological processes without properly active AGP [13, 19, 20].

The most current definition of AGP includes more specific features of AGP only. AGPs are defined as glycoproteins with the following characteristics: a protein domain rich in proline, alanine, serine, and threonine residues (PAST) with Ala-Hyp, Ser-Hyp, Thr-Hyp dipeptide repeats, the presence of type II AGs attached to Hyp residues, the occurrence of an N-terminal hydrophobic secretion signal sequence, the ability to attach to the plasma membrane by a C-terminal GPI anchor, and the ability to bind to the β-Yariv reagent, which recognizes the β-1,3-Gal main chains of type II AGs in the AGP molecule [7, 14].

The use of immunocytochemical approaches with antibodies recognizing specific epitopes of AGPs [11, 21–23] has shown that AGPs are common in plants [13]. Their presence is observed both in terrestrial and aquatic plants. Although AGPs occur in a low amount in the plant extracellular matrix, in comparison to pectins or other polysaccharides, they take part (individually or collectively) in numerous fundamental processes. Their role in the developmental process includes their involvement in somatic embryogenesis, embryo, and postembryonic pattern formation, male and female gametophyte development, advanced phases of microsporogenesis and megasporogenesis, pollen tube elongation, pollen tube movement along the transmission tissue during the progamic phase, double fertilization [24–27]. Also, the function of AGPs in morphogenesis is well known; from contribution to secondary cell wall deposition, and xylem differentiation to involvement in the growth of plant organs [11, 28–31]. Another significant AGPs participation in plant physiological programmes is the contribution to root-pathogen interactions, where the occurrence of AGP-rich rhizodeposits improves plant condition [29]. The AGPs up-regulation by pathogen attack is correlated with their role in plant susceptibility as signaling molecules enhancing cell-cell communication [32]. Moreover, the release of AGPs under high salinity stress may maintain cell wall integrity, because the turnover of AGPs and minimal Ara content probably is required for proper root elongation by regulation of root cell orientation. Furthermore, the secretion of AGPs under high salinity is connected with the reduction of Na + activity and may attenuate ion toxicity in plants [33]. Overall, up-regulated and down-regulated level of AGPs as a response to unfavorable environmental conditions is connected with the rigidification of the cell wall and the formation of a protective zone in the extracellular plant matrix [32, 34]. Nowadays, it is assumed that these AGP roles are likely related to the unique AGP structure that allows interactions with other cell wall-plasma membrane components [30, 35, 36].

### Structure of AGPs

Briefly, the basic structure of AGP is an amino acid core mainly composed of a PAST region with attached carbohydrate chains, which are anchored in the plasma membrane via glycosylphosphatidylinositol anchor (GPI) [37–39].

#### Protein backbone

The protein backbones vary widely and, in classical AGPs, have a length of approximately 100 amino acid residues. The amino acids in the protein backbone are typically arranged in repeating dipeptide motifs: Ala-Pro, Ser-Pro, Thr-Pro, and Val-Pro. The Pro residues are transformed into Hyp by a multigene family of enzymes in the Pro hydroxylation process [40, 41]. Most AGP protein backbones have an N-terminal secretion signal and a C-terminal hydrophobic domain to which GPI anchors can be attached. Probably, the core protein is just a scaffold for attaching sugar residues that directs the side-chain assembly of oligo- and polysaccharides to select regions along the backbone [42, 43].

#### GPI anchor

There are reports that some AGPs have a distinctive feature namely a GPI anchor, which is determined by a phosphoceramide lipid composed of phytosphingosine and tetracosanoic acid, and a partial β-galactosyl substitution of its core oligosaccharide [11]. However, research on *Arabidopsis* identifies 55 of the 85 AGPs that contain GPI anchors [39]. Thus, this feature is rather attributed to Classical AGPs. The GPI anchor binds to a specific amino acid in the C-terminal part of AGP, thereby removing the C-terminal hydrophobic tail. The process takes place in the ER and allows the anchoring of the AGP to the ER membrane and to the Golgi apparatus. Moreover, the AGP molecule can be cleaved by phospholipase C (PLC) and phospholipase D (PLD), and next released from the plasma membrane into the cell wall and the extracellular matrix [11, 44].

It has been suggested that the GPI-anchoring of the AGP molecule allows classifying AGPs as ‘GPI-anchored proteins’ (GAPs), which are regarded as essential elements of the cell wall determining its connections with the plasma membrane [45]. In addition, GAPs can bind to other components of the cell wall- plasma membrane, regulate its integrity and allow the formation of a continuum between both these external parts of the plant cell [45]. However, the exact role of GPI anchors in the functioning of AGPs is unknown. Some GAPs are involved in signal transduction by interacting with other proteins present in the plasma membrane. These interactions can occur with proteins of the same cell or with proteins of neighboring cells. GPI-anchored AGPs may be involved in signaling through disconnection of the protein from its anchor by phospholipase action, which may generate intra- and extracellular messengers [46].

#### Carbohydrate chains

AGP sugar chains account for over 90% of the total AGP molecular mass. The polysaccharide units vary in length (30–150 sugar residues) but retain the structure of arabinogalactan type II (AG II). Type II AG consists of a (1 → 3)-β-galactan core with attached (1 → 6)-β-galactan chains connected to each other by (1 → 3, 1 → 6)-linked branch points, O-3, and O-6 positions substituted with terminal arabinosyl residues [22, 37]. The side chains can be further modified with arabinose and other sugars, including L-rhamnose, L-fucose, D-glucosamine, D-mannose, D-xylose, D-glucose, D-glucuronic acid, and D-galacturonic acid [37, 47]. Polysaccharide chains are probably attached to Hyp, Ser, and Thr residues. Some AGPs have galactosyl-*O*-Hyp, galactosyl-*O*-Ser, arabinosyl-*O*-Hyp, and unidentified Thr-related glycosyl residues [11]. Furthermore, the use of NMR approaches facilitated the creation of a model of the Hyp-arabinogalactan polysaccharide from synthetic AG glycomodules, indicating possible glycosidic branch points. This allowed the formation of a glycan structure providing a contact surface for interactions with other molecules [28].

Research on the AGP molecule suggests that the protein moieties are only a scaffold for glycan attachment, and the AGP functions are directly based on glycosylation. In addition, the attachment of sugar chains influences the localization, intracellular distribution, and stability of AGPs. It is known that extensive glycosylation of the protein core makes AGPs more resistant to proteolysis [22]. Additionally, the varying level of glycosylation exerts an effect on molecular mass and determines the shape of the AGP molecule, as a result of which three models of the molecular structure have been proposed and described below [48, 49].

#### Molecular shape of AGPs

Imaging of AGPs using transmission electron microscopy showed two types of AGP molecular shapes: globular and rod-like. Nowadays, three models of AGP molecular organization have been proposed: the wattle-blossom, the twisted hairy rope, and the necklace [11, 18, 50]. In the wattle-blossom model, polysaccharide chains arranged in spherical units are anchored in the protein core to form a spheroidal particle. The twisted hairy rope model predicts that oligoarabinosides and AG chains wrap along the protein backbone [11]. The latest necklace model suggests that the AGP structure is a longitudinal AGP polypeptide ‘decorated’ by glycomodules [51].

### Synthesis of AGPs

The synthesis of the AGP molecule involves several steps that include the addition and removal of amino acids, lipids, and carbohydrates. Due to the high sugar content in AGPs, one of the main stages of their biosynthesis is glycosylation. Each of these post-translational modifications occurs on specific amino acid sequences and requires the presence of multiple enzymes [6, 52].

#### Proline-hydroxylation

Peptidyl Pro residues are mainly hydroxylated as the post-translation modification, which is catalyzed by prolyl 4 hydroxylases (P4Hs). Subsequently, the hydroxyproline (Hyp) residues are recognized as sites for glycans attachment in cell wall glycoproteins which are translocated via the secretory pathway [6].

The hydroxylation of Pro residues depends on certain amino acid motifs which were identified based on experimental data [6, 53, 54]. This Hyp code indicates that prolines are hydroxylated when are preceded by Val, Thr, Gln, Ser, Ala, Pro, Hyp [31]. All the other amino acids preceding the first Pro results in non-hydroxylation while the second one is hydroxylated [53]. However, extended variability was observed in Pro hydroxylation patterns while new patterns were observed in polypeptides not known before to be hydroxylated [6].

P4Hs belong to the large enzyme family of 2-oxoglutarate-dependent dioxygenases (2OGDs) and particularly to the class DOXB according to amino acid sequence similarities [55]. P4H activity requires molecular oxygen and 2-oxoglutarate as substrates, Fe^2+^ as a cofactor, and ascorbate for optimal activity [28, 56].

#### Glycosylation

The addition of carbohydrates to the protein backbone may change the physicochemical properties of the protein and its biological function [57]. The synthesis of AGP glycans engages at least 10 enzymes with different functions, e.g. galactosyltransferases (GALT), arabinosyltransferases, rhamnosyltransferases, fucosyltransferases (FUT), glucuronosyltransferases (GLCAT), xylosyltransferases, glucuronic acid methyltransferases, and glycoside hydrolases [57].

Glycosylation includes *N*-glycosylation, *O*-glycosylation, and glypiation [37].

*N*-glycosylation begins in the endoplasmic reticulum (ER) by the transfer of the oligosaccharide precursor to the amide nitrogen of Asn residues in specific Asn-X-Ser/Thr motifs where X can be any amino acid except Pro. During the transport of the glycoprotein along the secretory pathway, *N*-glycan undergoes transformations by adding and removing sugar residues in the ER and Golgi apparatus [57]. Glycosylation starts from the addition of the first Gal residue to the hydroxyl group of Hyp in the presence of galactosyltransferases (Hyp-*O*-GALTs). This step allows for the further addition of sugar chains and glycosyltransferases (GTs). The task of GT is to create glycosidic bonds between the carbohydrate residues and the acceptor molecule [40, 58]. Moreover, GTs act as regulators of the length, density, and sequences of AG chains [59].

*O*-glycosylation occurs mainly on Hyp residues in the Golgi apparatus, but sometimes it can also take place in other amino acids such as Ser and Thr residues [42]. Residues Hyp in AGPs can be glycosylated by two types of *O*-glycosylation: Hyp arabinosylation and Hyp arabinogalactosylation. Based on the contiguity theory, non-continuous Hyp residues are galactosylated by the addition of large 30–150 acid residues or neutral AG polysaccharides, and contiguous Hyp residues are arabinosylated by adding short 4–6 residue oligoarabinoside chains [41, 57]. Finally, type II AGs are *O*-glycosidically linked to the Hyp residues constituting the AGP carbohydrate domain as a greater part of a proteoglycan. It is known that the structure of type II AGs mainly consists of a β-(1 → 3)-galactan backbone substituted by β-(1 → 6) galactan side chains, which are modified with α-(1 → 3)-1-arabinofuranose and decorated with β-glucuronic acid [11, 60, 61].

The last step of AGP synthesis is glypiation, i.e. a post-translational modification allowing AGP to attach to the plasma membrane by the addition of glycophosphatidylinositol (GPI)-anchors [6]. GPI is attached at the C-terminal part of AGP. This region is made up of ~ 11 polar residues. It is followed by a ω region of ~ 4 small residues with a ω site, a spacer region of ~ 6 moderately polar residues, and a C-terminal hydrophobic region varying in length from 9 to 24 residues [7, 52, 62].

### Classifications of AGPs

According to the amino acid sequence, AGPs are divided into classical and non-classical AGPs [11, 18]. Classical AGPs have an N-terminal hydrophobic secretion signal sequence, a central domain containing PAST-rich regions, and a C-terminal hydrophobic sequence, i.e. the GPI anchor, which takes part in the attachment of AGPs to the plasma membrane [11, 18]. Non-classical AGPs also contain an N-terminal hydrophobic secretion signal sequence but are followed by one or more PAST-rich regions. These regions can be Hyp-*O*-glycosylated, including other non-PAST-rich regions, for example, C-terminal hydrophilic Asn-rich domains [18]. There are also non-classical chimeric AGPs, which are divided into three subfamilies: phytocyanin-like AGPs (PAGs), fasciclin-like AGPs (FLAs), and xylogen-like AGPs (XYLPs) [14, 31]. Furthermore, there are other AGPs that cannot be classified into any of these three families. One of them are AGPs referred to as chimeric AGPs with a protein backbone made of AGP motifs and non-HRGP motifs as well as hybrid AGPs, in which the protein backbone contains the characteristic motifs for AGPs and extensins [14].

### ARABINOXYLAN pectin arabinogalactan protein 1

Research shows that AGPs covalently associate with other components of the cell wall to form a dynamic ordering network of polysaccharides and proteoglycans in the cell wall-plasma membrane continuum called ARABINOXYLAN PECTIN ARABINOGALACTAN PROTEIN 1 - the APAP1 complex [3, 8, 30]. The APAP1 complex consists of an AGP as a core with attached classic type II AG, arabinoxylan, and pectin domains linked to Hyp in the polypeptide moiety. In turn, HG, RG-I, and short HG oligosaccharides constitute a pectic domain, which is linked to the Rha residue in the Rha-1 → 4-GlcA AG side chain. Arabinoxylan is directly attached to the Ara residue from the AG domain and to Rha in the RG-I domain [3, 8, 30].

The synthesis of the APAP1 complex can occur due to the attachment of pectin and arabinoxylan glycans to AGP in the extracellular matrix by endotransglycosylases, similar to xyloglucan remodeling in the cell wall [26]. Comparative glycome-profiling studies on APAP1 mutants versus wild-type *Arabidopsis* plants indicated reduced covalent linkages in the pectin fraction in stem cell walls [30, 63]. The existence of the APAP1 complex and its effect on cell wall properties confirm the hypothesis that AGPs act as a structural cross-linker in the cell wall and as a polysaccharide plasticizer [64, 65].

## Biosynthetic pathways of AGPs

**Fig. 1:**
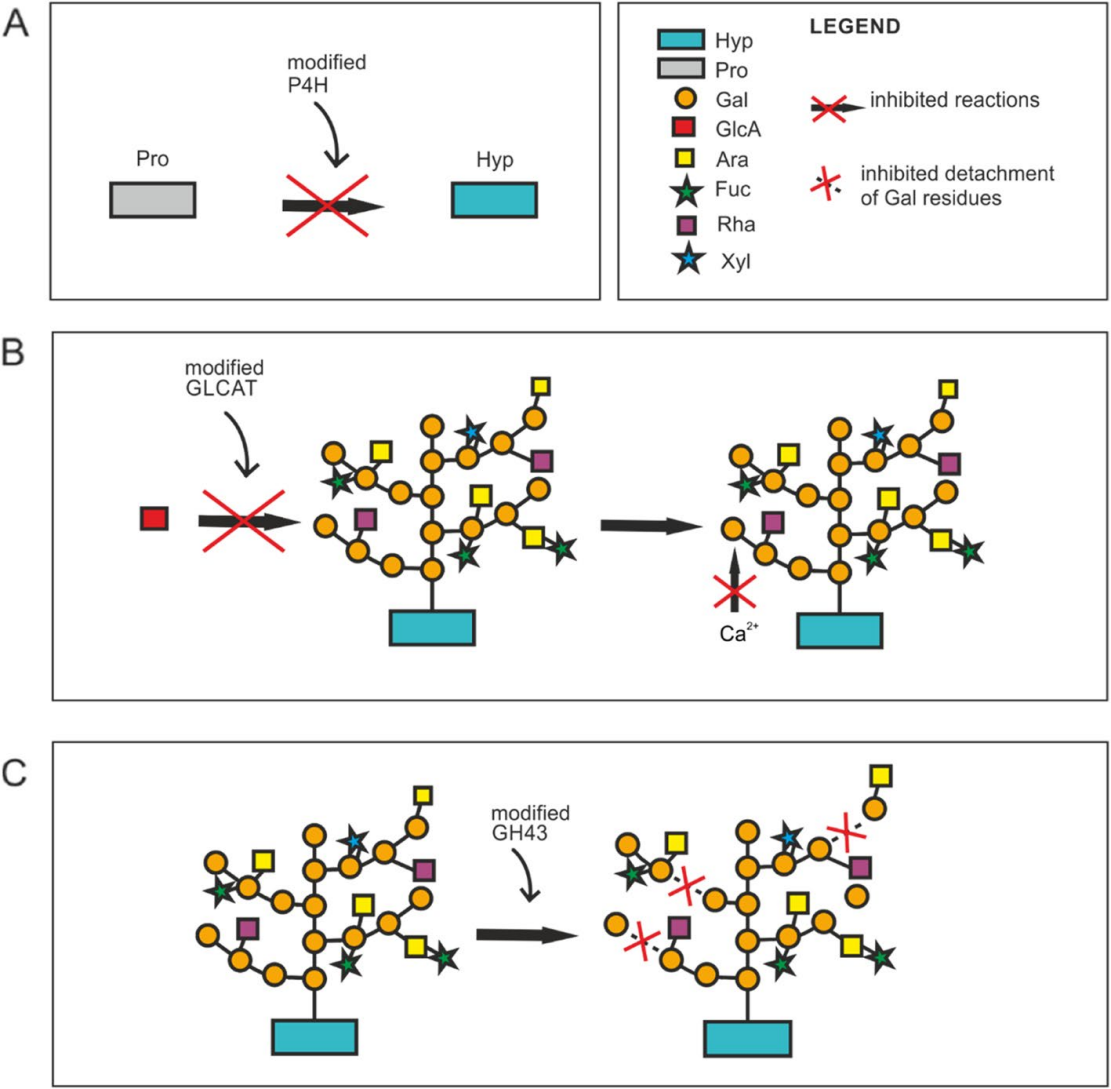
Schematic representation of described modifications in AGP synthesis: disturbance in Pro hydroxylation a, modification of GLCAT b, modification in GH43 activity c. Abbreviations in legend: Hyp – hydroxyproline, Pro – proline, Gal – galactose, GlcA – glucuronic acid, Ara – arabinose, Fuc – fucose, Rha – rhamnose, Xyl – xylose

Genetic modification of the biosynthesis process is used to elucidate the functional diversity of AGPs. Disruptions in the Pro hydroxylation and glycosylation processes by silencing or overexpressing specific genes probably induce changes in the plant cell wall and cell wall-plasma membrane connections (Fig. 1). Currently, various scientific reports describe the genetic modifications of the activity of enzymes responsible for proper AGP glycosylation. We focused on modification of Pro hydroxylation (*SlP4H1, SlP4H7, SlP4H9*), modification of GLCAT activity (*GLCAT14A, GLCAT14B, GLCAT14C, GLCAT14D, GLCAT14E*), and modification of GH43 activity (*GH43A, GH43B*), which are directly responsible for the metabolism of AGPs. Here, three different modifications exerting an effect on the plant at the molecular, cellular, to tissue/organ levels and on the whole plant physiology have been selected (Table 1).

**Table 1:** Compact overview of modifications of *P4H*, *GLCAT*, and *GH43* activities in *Arabidopsis thaliana* and their effects observed at molecular, cellular, and organ levels

Modification type/gene modulation		Modification effect			Ref.
		molecular level	cellular level	organ/organism level	
**P4H mutants** *SlP4H1 SlP4H7 SlP4H9* *AtP4Hs*	gene down-regulation	*•* tissue-specific expression *•* inactivity of P4Hs *•* inhibition of hydroxylation process *•* lower extent for AGP substrate	*•* localization in punctuated compartments (the Golgi stacks) *•* reduced Hyp content in root cell walls *•* changes in extensins level	*•* reduced root hairs length *•* reduced root hairs density *•* lower level of adaptation to hypoxia and anoxia conditions	[72, 74]
	gene overexpression	*•* up-regulation of P4H activity *•* higher expression of genes associated with hypoxia	*•* alters in the cell wall assembly *•* different fibrils orientation	*•* increased rate of root hairs elongation *•* increased density of root hairs *•* increased plant biomass	
**GLCAT mutants** *GLCAT14A GLCAT14B GLCAT14C GLCAT14D GLCAT14E*	gene down-regulation	*•* formation of premature STOP codons *•* lack of GLCAT activity *•* changes in AGP glycan biosynthesis *•* increased Gal and Ara in AGPs	*•* increased AGP content in specific plant organs (silique, leaf rosettes, stems, pods) *•* reduced amount of GlcA in cell wall *•* reduced Ca^2+^ binding *•* abnormal structure of the primary cell wall	*•* delayed seed germination and hypersensitivity to the stratification conditions *•* defective non-germinating pollen grain *•* reduced root hairs length *•* reduced plant height *•* reduction in adherent seed mucilage	[80]
	gene down-regulation	*•* lack of GLCAT activity *•* defective synthesis of AGs *•* reduction of glucuronidation of AGs *•* elongation of the branched β-1,3- and β-1,6-galactan	*•* significant reduced Ca^2+^ binding capacity of AGPs (in comparison to the higher-order mutants and WT) *•* changes in intracellular Ca^2+^ transient signals *•* perturbed calcium waves in roots	*•* smaller trichomes and branching defect *•* crooked seedlings *•* shorter inflorescences *•* etiolated hypocotyl phenotype *•* limited seedling growth *•* plant growth deficiencies	[81]
**GH43 mutants** *GH43A GH43B*	gene down-regulation	*•* lack of *GH43* β-1,3-galactosidase activity *•* inaccessible of β-1,3-linkages *•* disruption in the glycosylation process *•* changes in AGP glycan biosynthesis	*•* changes in the abundance of cell wall-associated AGPs *•* alters in the cell wall extensibility *•* stronger binding of certain AGPs to the cell wall *•* changes in pectin content (HG, RGI) *•* changes in cell wall polymer deposition	*•* root cell expansion defects *•* loss of anisotropic root growth *•* swelling of the root epidermis cells	[84]

### Modifications of AGP pro hydroxylation - P4H (prolyl 4 hydroxylase) mutants

Membrane-bound P4Hs belong to the family of 2-oxoglutarate-dependent dioxygenases catalyzing Pro hydroxylation, which is the major post-translational modification of AGPs. This process takes place prior to *O*-glycosylation to define the subsequent sites of the attachment of sugar chains [66]. Research shows that proper Pro hydroxylation is essential for cell differentiation and plant development. The use of inhibitors of P4H activity, such as 3,4-dehydroproline (DHP), which is a proline analogue, inhibits hydroxylation in carrot roots in vivo [67]. Pyridine 2,4-dicarboxylate (PDCA) is also a 2-oxoglutrate structural analogue, a known inhibitor of 2-oxoglutarate dependent dioxygenases, and a potent inhibitor of the in vitro enzymatic activity of two carnation recombinant P4Hs, DcP4H1 and DcP4H2 [68]. Application of PDCA in cut carnation flowers suppressed climacteric ethylene production and as a result delayed senescence indicating a correlation of proline 4 hydroxylase activity with ethylene production. Moreover, differential *Km* values of the recombinant DcP4H1 and DcP4H2 proteins for synthetic peptides representing plant AGPs and extensins suggested specificity of P4H activity for substrate proteins [68]. However, in a recent report, all four tobacco P4Hs could hydroxylate proline residues in synthetic peptides of two human and two plant proteins without exhibiting specific preferences indicating similarities in substrate specificity [69].

Moreover, PDCA induced shorter roots and hypocotyls in tomato seedlings which were accompanied by a decrease in hydroxyproline content and in AGP-bound epitopes in a dose-dependent manner [70]. A similar reduction in AGP-bound epitopes and ethylene production was obtained when PDCA was applied on tomato pericarp discs of two ripening stages indicating possible suppression of P4H enzymatic activity by this 2-oxoglutarate analogue [71].

Arabidopsis P4Hs T-DNA knock out mutants showed a short root hair phenotype with reduced density while in overexpression lines the hair length doubled and the hair density increased indicating that Pro hydroxylation is required for root hair cell elongation [66]. These results demonstrated the AGPs and extensins function in the cell wall assembly through interactions with other polysaccharides [66].

Studies on multiple P4H T-DNA knock out lines, including localization analysis, BiFC Protein-Protein interaction, FRET, phenotypic analysis, and TEM cell wall imaging, revealed that overexpression and silencing of *P4H* genes lead to changes in the *O*-glycosylation process, resulting in an altered synthesis of the AGPs [72]. Velasquez et al. [72] confirmed that the catalytic activity of P4H5 is required for root hair tip expansion through involvement in AGP *O*-glycosylation. Moreover, microscopic studies on structural differences in the cell wall architecture in deficient proline hydroxylation and subsequent *O*-glycosylation Arabidopsis lines, in comparison to wild type plants, highlighted the alterations in the orientation of fibrils in root hair tips. Fibrils observed in the *p4h* mutants appeared at angles between + 20° and/or − 20°, while in wild type was oriented at + 60° and/or − 60°. These results suggest that improper hydroxylation leads to disturbances in the cell wall structure and, consequently, to a change in the proper course of physiological processes, such as cell differentiation [72].

Moreover, the *Arabidopsis* AGP21 peptide which is hydroxylated and *O*-glycosylated is induced by the brassinosteroids BZR1 transcription factor to regulate root hair development through suppression of the homeodomain protein GLABRA 2 (GL2) [73]. These results indicate the significance of Pro hydroxylation and subsequent *O*-glycosylation for proper subcellular localization and function of AGPs.

P4Hs, as members of the large family of 2-oxoglutarate-dependent dioxygenases, require the presence of molecular oxygen to catalyze proline hydroxylation of HRGPs. A T-DNA knock out mutant of *Arabidopsis* P4H3 (AtP4H3) showed a lower survival rate under anoxic conditions [74]. This decreased tolerance to anoxia was accompanied by lower AGPs- and higher extensins-bound epitopes, which were associated with *AtP4H3* inactivation suggesting possible involvement in stress adaptations [74]. This decrease in AGPs-bound epitopes might be related to the altered frequency of proline hydroxylation occurrence due to the suppression of *AtP4H3* enzymatic activity [74].

Furthermore, suppression of the P4H expression causes various morphological changes in tomato plant organs [75]. To understand the P4H role in plant development and growth, virus-induced gene silencing (VIGS) was used using the tobacco rattle virus (TRV). Three tomato *P4H* genes were silenced, *SlP4H1*, *SlP4H7*, and *SlP4H9* [75]. The expression of *SlP4H1*, *SlP4H7*, and *SlP4H9* was clearly suppressed in the shoot while only the *SlP4H7* and *SlP4H9* expression was decreased in the roots, which was also associated with a reduction of the total hydroxyproline content. Immunocytochemical studies on lines with silenced *SlP4H7* and *SlP4H9* using JIM8 and JIM11 antibodies showed reduced content of AGPs and extensins in shoots, stems, and leaves, indicating that tomato P4Hs are involved in the synthesis of AGPs and extensins [75]. The VIGS-plants were characterized by longer shoots and a larger leaf area, suggesting that these modifications led to an increase in the intensity of cell division and cell expansion. VIGS-leaves with a partially silenced *SlP4H7* transcript had epidermal cells with increased surface area and an unchanged number of leaf cells with a less spherical shape. VIGS lines with a partially silenced *SlP4H9* mRNA showed an increased number of cells with a rounder shape than in the VIGS-*SlP4H7* cells. This suggests that P4Hs are involved in the morphology of leaf epidermis cells and induced changes in the rearrangement of xyloglucan and pectins [75].

Stable suppression of *S1P4H3* transgenic lines using an RNAi approach was the basis for studies on P4H involvement in the regulation of tomato fruit abscission zone (AZ) [76, 77]. The mutant lines were characterized by alterations in the AZ zone morphology and distal pedicel length as well as changes in the flower and fruit abscission process. Moreover, the up- and down-regulation in *S1P4H3* gene expression levels induced changes in the content and structure of AGPs and pectins in AZ and the proximal and distal pedicel [76, 77]. The silencing of *SlP4H3* shifted the AZ closer to the subtending organ, while the overexpression was further apart [76]. This was attributed to the shorter distal part of the pedicel in the RNAi lines and the longer in the OEX lines which was also related to the intensity of parenchyma cell division [76]. In addition, the *SlP4H3* silencing in three RNAi lines delayed the natural abscission of overripe tomato fruits 90 days after the breaker stage which was correlated to reduced expression of abscission-specific cell wall hydrolases such as TAPG2, TAPG4 (encoding polygalacturonase genes) and CEL2 (gene encoding cellulase) [76, 77]. Ethylene-induced flower abscission was accelerated in the RNAi lines and delayed in the OEX mutants [76]. The results indicated that P4Hs regulate the molecular and morphological features of the abscission zone and distal pedicel cell walls by involvement in AGP synthesis. In turn, alterations in the AGP structure have an influence on the progression of flower abscission and directly affect the regulatory mechanism of fruit abscission.

### Modifications of AGP glycosylation – GLCAT (β-glucuronosyltransferase) mutants

Glucuronic acid (GlcA) is attached to the carbohydrate moieties of AG and, more specifically, to the ends of the β-(1 → 6)-galactan side chains [60]. The localization of GlcA suggests that glucuronidation processes may have a large impact on AGP properties, such as the ability to bind Ca^2+^ ions [78] or elongation of β-(1 → 6)-galactan side chains during the biosynthesis process [79].

*Arabidopsis* β-glucuronosyltransferases, including GLCAT14A, GLCAT14B, and GLCAT14C belonging to the family of glycosyltransferases 14 (GT), are responsible for the transfer of GlcA residues to the AGP molecule. *GT* genes are highly redundant; therefore, in order to fully understand the functions of the sugar moiety attached to AGP, it is necessary to create higher-order mutants, in which many genes from this gene family are deactivated. For comprehensive studies, multiple gene knockout mutants were generated by Zhang et al. [80]. This was possible due to the CRISPR-Cas9 multiplexing approach, which allows the generation of mutations in multiple genes in a single transformation process by designing RNAs (gRNAs) targeting multiple *GT* genes. The function of GlcA residues in AGP with emphasis on plant growth and development was assessed with the use of in silico analyses, AGP quantification, monosaccharide composition analysis with HPAEC-PAD, calcium-binding assays, morphological observations of root hair and trichomes, and an in vitro germination experiment. It is important to note that GlcA is the only sugar in the AGPs glucan part which is negatively charged.

Quantitative analysis of glycosylated AGP carried out using the β-Yariv reagent in *glcat* mutants showed a higher amount of AGP, an increased amount of Gal, and a decreased amount of GlcA. Also, the triple mutants were characterized by a more pronounced reduction of GlcA than the double mutants. These results indicated that *GLCAT14A* and *GLCAT14B* play redundant roles in catalyzing the transfer of GlcA to AGP [80].

Detection of calcium associated with AGPs revealed a reduction of calcium binding in *glcat* mutants, in comparison with WT. These observations suggest that GlcA is responsible for calcium binding, and the loss of *GLCAT* genes is connected with a significant reduction in AGP calcium binding. The alterations observed at the cellular level were reflected in delayed germination, reduction in adherent seed coat mucilage, reduced trichome branching, and reduced root hair length. The double and triple mutants showed less intensive plant growth, suggesting that the GlcA residues on AGP are involved in cell differentiation and tip-focused growth. Presumably, these changes are related to the lower levels of GlcA in the AGP and hence the reduced calcium influx into particular tissues [80].

Zhang et al. [80] suggested a mechanism that may be responsible for all these changes, i.e. calcium-mediated signaling combined with cell wall structural interactions. Namely, it was assumed that GlcA residues in AGPs served to terminate the elongation of β-1,3- and β-1,6-galactan sugar additions to AGPs. Thus, the lack of GlcA is a cause of the incorporation of the β-1,3-galactose backbone and β-1,6-galactose side chains in AGPs. Although they are present in AGPs and in the whole cell in small amounts, GlcA residues may affect the interaction with other components of the cell wall. Precise analyses show that GlcA is a covalent linker between AGP and pectin in the APAP1 complex, which is important for proper cell wall assembly. Such structural interactions are correlated with controlling the stiffness and extensibility of the cell wall. Therefore, the reduction of GlcA in AGPs can disrupt the substantial structure of the primary cell wall and impair the release of pectin to form proper seed coat mucilage [80].

Investigation of the biological role of glucuronidation of AG polysaccharides was the aim of the study performed by Lopez-Hernandez et al. [81]. The basis for the study was the mutant generation with reduced activity of *GLCAT: GLCAT14A, GLCAT14AB,* and two genes *GLCAT14D* and *GLCAT14E*, which were characterized by the highest expression in leaves and roots. The changes observed in the plant phenotypes were indisputably connected with the lower amount of GlcA in AGs, which had consequences for the AGP-Ca^2+^ interaction. Studies of AGP extracted from rosette leaves and roots of single and double *glcat14a/b* mutants showed a significant reduction in the amount of glucuronidated galactose residues. Moreover, the level of glucuronidated Gal in AGP extracted from the *glcat14a/b* roots decreased below the level noted in the *glcat14a* mutant. Thus, it is undeniable that both *GLCAT14A* and *GLCAT14B* are involved in the glucuronidation process. Also, in vitro studies with *glcat14d-1* and *glcat14e* showed a significant reduction in the amount of glucuronidated Gal in the leaf AGP compared to WT. It is therefore likely that *GLCAT14D* and *GLCAT14E* are responsible for the glucuronidation of the longer AGP sugar chains. In order to study the *GLCAT14* activity in more detail, Lopez-Hernandez et al. [81] generated triple mutants: *glcat14a/b/d* and *glcat14a/b/e.* In the case of the first mutant, there was a significant decrease in glucuronidated Gal in both leaves and roots, compared to the single and double mutants. The production of a liquid culture of the glcat14a/b/e and glcat14a/b callus used as a reference confirmed the decrease in the level of AG glucuronidation.

Furthermore, Ca^2+^ binding capacity tests in triple mutants with reduced glucuronidation showed a significant reduction (80%) of Ca^2+^ binding compared to WT plants. Therefore, it can be concluded that glucuronidation is necessary for the binding of Ca^2+^ to AGP. The generation of double and triple mutants, in which a decrease in the level of glucuronidation was noted, revealed numerous developmental defects. Moreover, the higher-order mutants were characterized by a defect in the growth and branching of trichomes. All these data confirm that AGP glucuronidation is extremely important for the proper development of plants [81].

The defects in the development of *glcat14* mutants are probably caused by the reduced Ca^2+^ binding capacity. In addition to AGPs, homogalacturonan is responsible for Ca^2+^ binding in the cell wall [82]. Glucuronic acid in AGPs is a covalent linker between AGP and pectin in the APAP1 complex [30], which is important for the synthesis and correct structure of pectin in the cell wall. Lopez-Hernandez et al. [81] put forward a hypothesis that *glcat14* mutants may be pectin defective, but their assumptions have not been confirmed. It led to numerous molecular and cellular changes connected with deficiencies in the spatiotemporal propagation of Ca^2+^ waves and, consequently, in intracellular Ca^2+^ signaling. The authors proposed a hypothesis that the GlcA residue of AGPs which is a covalent link between AGP and pectin in APAP1, may affect cell wall integrity and exert an effect on cell shape formation and expansion. Based on these findings, Lopez-Hernandez et al. [81] suggested that an increase in the extracellular Ca^2+^ concentration is correlated with the release of ions from AGP-Ca^2+^. The proposed mechanism may be mobilized by local activation of H^+^ − ATPases or by the recruitment of Ca^2+^ channels. Overall, all alterations at the molecular and cellular levels had an influence on multiple developmental processes in plants.

### Modifications of AGP glycan hydrolyzing activity - GH43 (glycoside hydrolase family 43) mutants

According to the carbohydrate-active enzyme database, glycoside hydrolase family 43 (*GH43*) is a group of enzymes involved in cell wall biosynthesis; they can also affect the AGP glycosylation process. Two genes *GH43A* and *GH43B* from the *GH43* family were found in the *Arabidopsis thaliana* genome. They are expressed in the cell elongation zone above the root meristem [83]. The characterization of their potential function in AGP glycan hydrolyzing activity was described by Nibbering and coworkers [84].

To understand the functional role of *GH43*, Nibbering et al. [84] created mutants carrying T-DNA exon insertions in *GH43A* and *GH43B*. They amplified the genomic sequences of *GH43A* and *GH43B* using specific primers. The amplified fragments were cloned into the pDONR207 plasmid via Gibson splicing and recombined into pHGY. Next, constructs were transformed into the *gh43null* mutant by transformation with *Agrobacterium tumefaciens*. The subsequent analyses of the mutants included *GH43* protein localization, heterologous expression of the *GH43* proteins, *GH43* protein activity assays, AGP quantification, and semiquantitative comprehensive microarray polymer profiling (CoMPP).

Morphological observations revealed almost complete inhibition of root growth and swelling of root epidermal cells in the *gh43null* mutant. Microscopic analyses performed with the use of yellow fluorescent proteins (YFPs) showed the subcellular localization of *GH43*, exactly in the elongation zone of young roots and in the root cap cells. The investigations of the presence of *GH43* substrates in the cell wall of recombinant *GH43* conducted using sequential extraction of glycoproteins revealed a lack of galactose in all obtained fractions and indicated that β-1,3-linkages were resistant to the *GH43* activity. More detailed analyses carried out at the molecular and cellular levels confirmed other differences between WT and the *gh43null* mutant. The defects in the AG glycosylation process in the mutant were connected with changes in the extractability of AGPs and in the content of AGPs associated with the cell wall. As proposed by Nibbering et al. [84], *GH43* is related to β-1,3-galactan processing during early AGP glycan modifications in the Golgi apparatus by regulation of the length of the β-1,3-galactan backbone. More detailed analyses of the *GH43* enzymatic mechanism of the synthesis of β-1,6-galactose with branched β-1,3-galactan oligosaccharides on the non-reducing and reducing β-D-Galp-(1 → 3)-β-D-GalpOMe ends showed that, when β-1,6-galactose is present at the non-reducing end, GH43 do not hydrolyze β-D-Galp-(1 → 3)-β-D-GalpOMe. This confirms that *GH43* in *Arabidopsis* is exo-β-1,3-galactosidase with strict substrate specificity. Probably, *GH43* is also involved in the modification of glycans by cutting off incorrectly synthesized β-1,3-galactans. The CoMPP results indicated that the lack of *GH43* β-1,3-galactosidase activity was the cause of all observed defects and correlated with changes in the abundance of other cell wall constituents. Decreased HG and RG-I content and an increased amount of β-1,4-galactan were noted in the *gh43null* mutant. The silencing of the *GH43* genes caused changes in the structure of the cell wall, which were reflected in changes in pectin extractability and suggested that the APAP1 complex and AGPs are the target of *GH43* activity.

A conclusion of the research performed by Nibbering et al. [84] was the statement that β-1,3-galactosidase affects the glycosylation process during AGP synthesis. The authors suggested the role of AGPs as chaperons ‘facilitating cell wall polymer deposition’, which may impair cell wall stiffening during cell expansion [84].

## Conclusion and research perspectives

Due to the unique structure, i.e. the presence of a specific protein domain, the extensive glycosylation, and the GPI anchor addition, AGP is certainly an enigmatic molecule. Moreover, the connections of AGPs with other cell wall components are crucial in the creation of a proper assembly and continuum between the cell wall and plasma membrane and the regulation of many mechanisms in plants. Despite its small amounts in the extracellular matrix, it is involved in many physiological processes related to plant growth and development.

The Hyp-*O*-galactosyltransferases enzymatic activity adds the first galactose molecule on the Hyp residue initiating the AGPs glycan backbone for further glycans molecules addition. Therefore, this step is considered the next proline hydroxylation step in the biosynthesis of the glycan part of AGPs. In this context, high order *Arabidopsis* knock out mutants of Hyp-GALTs [85], to bypass their redundant function, might provide phenotypes that might resemble those of P4H mutants. However, the current data do not support this assumption at least in *Arabidopsis* growth and development programmes.

The specific spatiotemporal localization, assembly, crosslinking, and loosening of cell wall components drive morphogenesis and maintenance of the correct shape of individual cells and thus cause changes at the molecular, cellular, and organ levels and in the whole plant homeostasis. This information allows a hypothesis that the absence of properly synthesized AGP would impose changes in mutual interactions with the surrounding environment. Nevertheless, there are no convincing models yet for the in vitro mechanisms of cell wall assembly. Despite the recent progress in understanding the role and functions of AGPs in plants, the effect of chemical changes in specific cell wall polymers on their roles is still unknown. Available knowledge allows for the formulation of subsequent important questions: Does the lack of AGPs disturb the proper assembly of the extracellular matrix? (1), Are the changes in AGPs distribution dependent on tissue type or developmental stage? (2), What triggers AGPs ‘migration’ along the matrix space? (3), Does calcium ion flux influence the functional mechanism of AGPs? (4). This information is still incomplete and further in-depth research is needed.
